# Arginine-Ornithine Antiporter ArcD Controls Arginine Metabolism and Interspecies Biofilm Development of Streptococcus gordonii*[Fn FN3]^♦^

**DOI:** 10.1074/jbc.M115.644401

**Published:** 2015-06-17

**Authors:** Akito Sakanaka, Masae Kuboniwa, Hiroki Takeuchi, Ei Hashino, Atsuo Amano

**Affiliations:** From the ‡ Department of Preventive Dentistry, Osaka University Graduate School of Dentistry, Suita, Osaka 565-0871 and; § CREST, Japan Science and Technology Agency, Saitama 332-0012, Japan

**Keywords:** biofilm, cell metabolism, metabolic regulation, metabolomics, Streptococcus, symbiosis, Fusobacterium, arginine metabolism, cross-feeding, ornithine export

## Abstract

Arginine is utilized by the oral inhabitant *Streptococcus gordonii* as a substrate of the arginine deiminase system (ADS), eventually producing ATP and NH_3_, the latter of which is responsible for microbial resistance to pH stress. *S. gordonii* expresses a putative arginine-ornithine antiporter (ArcD) whose function has not been investigated despite relevance to the ADS and potential influence on inter-bacterial communication with periodontal pathogens that utilize amino acids as a main energy source. Here, we generated an *S. gordonii* Δ*arcD* mutant to explore the role of ArcD in physiological homeostasis and bacterial cross-feeding. First, we confirmed that *S. gordonii* ArcD plays crucial roles for mediating arginine uptake and promoting bacterial growth, particularly under arginine-limited conditions. Next, metabolomic profiling and transcriptional analysis of the Δ*arcD* mutant revealed that deletion of this gene caused intracellular accumulation of ornithine leading to malfunction of the ADS and suppression of *de novo* arginine biosynthesis. The mutant strain also showed increased susceptibility to low pH stress due to reduced production of ammonia. Finally, accumulation of *Fusobacterium nucleatum* was found to be significantly decreased in biofilm formed by the Δ*arcD* mutant as compared with the wild-type strain, although ornithine supplementation restored fusobacterium biovolume in dual-species biofilms with the Δ*arcD* mutant and also enhanced single species biofilm development by *F. nucleatum*. Our results are the first direct evidence showing that *S. gordonii* ArcD modulates not only alkali and energy production but also interspecies interaction with *F. nucleatum*, thus initiating a middle stage of periodontopathic biofilm formation, by metabolic cross-feeding.

## Teaser

**Background:** Arginine and its derivatives are key factors for inter-bacterial communication in periodontal microflora.

**Results:** ArcD-mediated export of ornithine by oral commensal *Streptococcus gordonii* facilitates biofilm development of *Fusobacterium nucleatum*.

**Conclusion:** Ornithine cross-feeding from *S. gordonii* to *F. nucleatum* mediates dental biofilm maturation.

## Introduction

Periodontal disease is a polymicrobial infectious disorder that leads to inflammatory destruction of tooth-supporting tissues and the most common cause of tooth loss worldwide (1, 2). The pathogenesis involves dental biofilm development that is divided largely into three stages mediated by early, intermediate, and late colonizing microbes (3–5). Early colonizers include *Streptococci* and *Actinomyces*, which adhere to salivary pellicles on teeth by adhesins such as fimbriae and polysaccharides (3, 6–8). *Fusobacterium nucleatum*, which expresses multiple adhesins, is an intermediate colonizer that serves as a central bridging organism by selective co-aggregation with various early and late colonizers (9–12). This bacterium also reduces the level of oxygen and produces ammonia, thus providing a favorable biofilm environment for anaerobic late colonizers, including periodontal pathogens such as *T. denticola* and *P. gingivalis* (9, 13, 14). *P. gingivalis* generally plays a major role in the periodontal etiology, whereas a subset of oral streptococci, including *S. gordonii*, traditionally considered to be commensals, has recently been proposed to be “accessory pathogens” that are able to enhance the virulence of periodontal pathogens via microbial interactions in the heterotypic community (15). Interactions between oral streptococci and these pathogens include quorum sensing, cell surface adhesion-mediated co-aggregation, and metabolic cooperation. Recently, interest has been growing in regard to the metabolic cooperation of *S. gordonii*, because this species was shown to have metabolic compatibility with *F. nucleatum* and *P. gingivalis* in multispecies biofilm communities, likely contributing to periodontal destruction (5, 16).

An increasing number of studies have shown that interactions between oral streptococci and other bacterial species involve arginine and arginine-derived metabolites. For example, co-aggregation with *Actinomyces oris* enables *S. gordonii* to survive in an arginine-restricted condition by stabilizing streptococcal arginine biosynthesis, suggesting that arginine has a vital role in interaction between these species (17). As for *Streptococcus intermedius*, its extracellular component arginine deiminase (ArcA) suppresses expression of the fimbrial subunit (FimA) of *P. gingivalis*, leading to diminished biofilm formation (18). A more recent report also suggested that FimA down-regulation can be attributed to the enzymatic activity of extracellular ArcA, which catalyzes the conversion of arginine to citrulline, implying that environmental arginine depletion has a negative effect on FimA expression (19). Additionally, *F. nucleatum* possesses an arginine-inhibitable adhesin (RadD) that is responsible for its adherence to oral streptococci (11). Furthermore, a recent metagenomic study found that genes encoding enzymes associated with arginine metabolism exhibited higher abundance in subgingival biofilms obtained from periodontal patients (20). Together, these findings indicate that arginine and its derivatives play key roles in streptococcal interactions with periodontal bacteria.

Arginine has also been implicated in the prevention of dental caries, because bacterial catabolism of arginine yields urea and ammonia, both of which contribute to the neutralization of biofilm acid (21–23). Specifically, the arginine deiminase system (ADS)^3^ of oral streptococci has been extensively studied as a major source of ammonia generation in oral microbial communities (24–27). This system catalyzes the conversion of arginine to ammonia and CO_2_, along with concomitant production of ATP with three core enzymes: ArcA, ArcB (catabolic ornithine carbamoyltransferase), and ArcC (carbamate kinase) (28). The *arcABC* genes are generally arranged in an operon, and their expression is considered to not only protect microbial communities from acid stress through ammonia production but also function as part of an energy-generating system (21). *S. gordonii* has been described as one of the few ADS-positive organisms in the oral cavity, and its regulation of ADS genes has been closely examined (24–27).

*S. gordonii* harbors the gene encoding the arginine-ornithine antiporter (ArcD), which is commonly located in the same locus with the *arcABC* genes. ArcD, a transmembrane protein, mediates the uptake of arginine and the concomitant export of ornithine in an ATP-independent manner, providing a substrate for the ADS pathway (29–32). Recent proteomic analyses revealed that the abundance of *S. gordonii* ArcD was decreased in model communities with *F. nucleatum* despite an increase in ArcABC, although no significant change was induced by co-incubation with *P. gingivalis* (33). Furthermore, co-aggregation with *A. oris* resulted in decreased expression of *arcD* in *S. gordonii* as compared with its planktonic state (17). These results indicate that ArcD is involved in bacterial metabolic interactions in *S. gordonii*. However, despite its relevance to the ADS and potential impact on microbial communities, ArcD of *S. gordonii* has yet to be examined in detail.

In this study, we generated an *S. gordonii* DL1 Δ*arcD* mutant and characterized the consequences of *arcD* mutation to determine its physiological role, as well as explore the possible involvement of ArcD in interactions with other bacteria. Our findings show that *S. gordonii* ArcD plays crucial roles for arginine uptake and ornithine excretion, and ArcD-mediated ornithine export contributes to maintaining the homeostasis of arginine metabolism. We also found that ornithine released by ArcD of *S. gordonii* facilitates dual-species biofilm formation with *F. nucleatum*. To the best of our knowledge, this is the first report of metabolic cross-feeding between *S. gordonii* and *F. nucleatum*.

## Experimental Procedures

### 

#### 

##### Bacterial Strains and Growth Conditions

The strains and plasmids used in this work are listed in Table 1. *S. gordonii* strains were grown statically at 37 °C in Todd-Hewitt broth (THB) or, when necessary, in chemically defined medium (CDM), which was developed according to a previously reported method (34) without the carbon source. Briefly, CDM has the following composition: 58 mm K_2_HPO_4_; 15 mm KH_2_PO_4_; 10 mm (NH_4_)_2_SO_4_; 35 mm NaCl; 0.2% casamino acids; 100 μm MnCl_2_·4H_2_O (pH 7.4); 2 mm MgSO_4_·7H_2_O; 0.04 mm nicotinic acid; 0.1 mm pyridoxine-HCl; 0.01 mm pantothenic acid; 1 μm riboflavin; 0.3 μm thiamine-HCl; 0.05 μm d-biotin; 4 mm l-glutamic acid; 1 mm l-arginine-HCl; 1.3 mm l-cysteine-HCl, and 0.1 mm l-tryptophan. For quantitation of the ADS intermediate in culture supernatants and biofilm assays, 1.2 and 0.025% glucose, respectively, was added to CDM as a carbon source. For ammonia assays, as well as metabolomics and transcriptome analyses, CDM with 0.1% galactose was used. *F. nucleatum* subsp. *nucleatum* ATCC 25586 and *P. gingivalis* ATCC 33277 were grown as described previously. For examining *F. nucleatum* planktonic growth, various concentrations of ornithine were added to growth medium containing yeast extract (10 g/liter), trypticase peptone (10 g/liter), biosate peptone (10 g/liter), brain heart infusion broth (19.2 g/liter), CaCl_2_ (72 μm), MgSO_4_ (66 μm), K_2_HPO_4_ (0.23 mm), KH_2_PO_4_ (0.29 mm), NaHCO_3_ (4.76 mm), NaCl (1.37 mm), hemin (5 mg/liter), and menadione (1 mg/liter). When necessary, the following antibiotics were used as selective markers for *S. gordonii*: erythromycin (5 μg/ml) and spectinomycin (100 μg/ml).

**TABLE 1:** **Bacterial strains and plasmids used in this study**

Strains and plasmids	Description	Source or Ref.
**Strains**		
*Streptococcus gordonii*		
DL1 Challis	Wild type	Pakula and Walczak (62)
Δ*arcD*	*arcD*::*ermAM* (ArcD^−^ Em^r^)	This study
Comp *arcD*	*arcD*::*ermAM*::pCK*arcD* (ArcD^+^ Em^r^ Spcm^r^)	This study
*Fusobacterium nucleatum* subsp. *nucleatum* ATCC 25586	Wild type	American Type Culture Collection
*Porphyromonas gingivalis* ATCC 33277	Wild type	American Type Culture Collection

**Plasmids**		
pCK*arcD*	*S. gordonii arcD* in pCK; Spcm^r^ Kan^r^	This study

##### Mutagenesis

A PCR-based overlap-extension method was employed to construct the *S. gordonii* DL1Δ*arcD* strain (35). Briefly, a 686-bp fragment upstream of the *arcD* coding region was first amplified from chromosomal DNA by PCR (forward, 5′-CTGAGAAGAGCGGAGCAACT-3′; reverse, 5′-TATATTTTTGTTCATTGTTTTCTCCTCCTAAAATCTAACAA-3′), as well as a 684-bp downstream fragment (forward, 5′-AACGGGAGGAAATAAGAAATGTGAGGTGCTTCCA-3′; reverse, 5′-CTCTAAGAAAGGCCCCTGGT-3′). The resultant amplicons were connected in a second step using long flanking-region PCR with a DNA fragment encoding an erythromycin resistance gene, *ermAM*, obtained from pVA891 (forward, 5′-TAGGAGGAGAAAACAATGAACAAAAATATAAAATATTCTCAAAAC-3′; reverse, 5′-AGCACCTCACATTTCTTATTTCCTCCCGTTAAATAATAG-3′). The generated PCR products were then used for transformation of *S. gordonii*, as described previously (35). Replacement of *arcD* by *ermAM* in the mutant strain was confirmed by PCR and DNA sequencing analyses. Transformants were selected by growth on THB agar plates containing erythromycin at a final concentration of 5 μg/ml.

##### Complementation of DL1ΔarcD

To complement *arcD* deletion, a DNA fragment containing the entire *arcD* gene and additional upstream and downstream regions with EcoRI restriction sites at both ends was synthesized. The resulting product was digested with EcoRI and cloned into an EcoRI-predigested pCK plasmid. Following cloning of an aad9 cassette (spectinomycin-resistance gene) (36) into pCK with *arcD* and its contiguous regions via the PstI site, the resulting plasmid was used to transform *S. gordonii* DL1Δ*arcD*. Correct integration of recombinant pCK at the disrupted *arcD* locus by a Campbell-like single-crossover was confirmed by PCR and sequencing analyses.

##### Arginine Uptake Assay

An arginine assay previously described was used with slight modifications (37). To determine the time course of arginine uptake, *S. gordonii* DL1 and its derivative strains at the late exponential phase (*A*_600_, 1.0–1.2) in THB were harvested by centrifugation and washed twice in PBS, and then 4 × 10^8^ cells were aliquoted into 400 μl of PBS. The reaction was started by adding l-[^3^H]arginine (37 MBq/mmol) at a final concentration of 50 μm. Cells in 100-μl aliquots were collected on 0.22-μm filters at various times by passing 4 ml of PBS through the filters. Radioactivity on the filters was measured for 1 min using a liquid scintillation counter LS6500 (Beckman Coulter, Brea, CA). To perform a substrate specificity assay, structural analogues of arginine and control amino acids at 100-fold excess were added to the assay solution, and the rate of arginine uptake by *S. gordonii* WT was measured following 20 s of incubation. For determining *K_m_* and *V*_max_ values, cells were incubated with increasing arginine concentrations (1, 10, 25, 50, and 100 μm: 37 MBq/mmol at each concentration) for 1 min, and then radioactivity was measured as described above. *K_m_* and *V*_max_ values were calculated based on a Michaelis-Menten saturation curve with a nonlinear curve fit using ORIGIN 2015.

##### Analysis of Metabolites in Culture Supernatants

*S. gordonii* DL1 and its derivative strains were grown at 37 °C in THB under atmospheric conditions. At the late exponential growth phase (*A*_600_, 1.0–1.2), bacteria were harvested by centrifugation, washed twice with PBS, and then inoculated at an *A*_600_ of 0.1 in CDM containing 1 mm arginine and 1.2% glucose. Cultures were aerobically incubated at 37 °C, and growth was monitored by measurement of *A*_600_. Various arginine concentrations from 0 to 3 mm were also used for this experiment.

Concentrations of arginine, ornithine, and citrulline in culture supernatants were determined in triplicate using an ultrapressure liquid chromatography system (Acquity UPLC System; Waters, Milford, MA), coupled to a 2996 polydiode array detector operating at 260 nm. Samples were separated into components using a BEH C18 column (100 × 2.1 mm inner diameter, 1.7-μm particle size; Waters). Obtained data were processed with Empower 2 software (Waters).

##### Intracellular Metabolite Measurement

Thirty-ml cultures of *S. gordonii* DL1 and the Δ*arcD* mutant, initially adjusted to an *A*_600_ of 0.8, were grown anaerobically at 37 °C in CDM containing 1 mm arginine and 0.1% galactose in triplicate. At the late (8 h of culture) exponential growth phase, bacterial cells were harvested by centrifugation, washed twice with 1 ml of ultrapure water, and then immediately fixed with 1 ml of methanol containing the internal standard components. To remove protein, 2 ml of chloroform and 0.8 ml of ultrapure water were added to the samples, which were thoroughly mixed and centrifuged at 2300 × *g* for 5 min at 4 °C. The upper aqueous layer was then transferred to ultrafilter tips (Amicon Ultrafilter System^TM^) and centrifuged at 9100 × *g* for 120 min at 4 °C. Filtered material was dried under reduced pressure, followed by suspension in 50 μl of ultrapure water.

##### Metabolomics Analysis

Measurement of extracted metabolites was performed by capillary electrophoresis time-of-flight mass spectroscopy (CE-TOF-MS) with commercial electrophoresis buffer (H3301-1001 and I3302-1023; Human Metabolome Technologies (HMT), Tsuruoka, Japan), as described previously (38). Identification of metabolites and evaluation of the relative amounts were done using Master Hands (version 2.13.0.8.h; Keio University, Tsuruoka, Japan) with the HMT metabolite database. The relative amount of each metabolite was calculated with reference to the HMT internal standard material.

##### Quantification of mRNA Transcripts

Triplicate samples for extracting total RNA were cultured under the same conditions as for the metabolomics analysis. Bacteria harvested by centrifugation were immediately frozen and homogenized with zirconia beads at 1500 rpm for 5 min at 4 °C using Shake Master neo (BioMedical Science, Tokyo, Japan). Total RNA was isolated using TRIzol reagent (Life Technologies, Inc.) and an RNeasy kit (Qiagen) and then reverse-transcribed into cDNA using iScript master mix (Bio-Rad), according to the manufacturer's protocol. Real time RT-PCR was performed with a KAPA SYBR Fast kit (KAPA Biosystems). Primers used are listed in Table 2. To assess gene expression, a comparative *C_t_* method was used.

**TABLE 2:** **Oligonucleotides used for real time RT-PCR**

Open reading frame	Targeted gene	Direction*^a^*	Sequence (5′–3′)	Product size	Source or Ref.
				*bp*	
SGO1593	*arcA*	F	GAAGCAGCAAAAGGCTTGAC	95	This study
		R	GCAAATGGATCTCGTGTGAA		
SGO1592	*arcB*	F	AGTTTTGGGCCGTATGTTTG	111	This study
		R	TCGTCAGTCAAACCATTCCA		
SGO1591	*arcC*	F	CATCTGATCCGTCAGCAAAA	114	This study
		R	GAGGACCATTCCCGTGAGTA		
SGO1569	*argC*	F	CCAGCCATTTTGTTCATGTG	89	This study
		R	CTGCAACTGCTGCACAATCT		
SGO1568	*argJ*	F	GACCAAAGCCTCAATCCAAA	111	This study
		R	TGCATCTCATAGGCAGCATC		
SGO1567	*argB*	F	GCCTTGCTAGGTCAAGTTGG	100	This study
		R	CCGAAACTGTCTTTCCCAAA		
SGO1566	*argD*	F	ATGCTGGAATTGATCCAAGG	82	This study
		R	GTTGGCAAAAATCAGCCAGT		
SGO0175	*argG*	F	AAACGATCAGGTCCGTTTTG	103	17
		R	GATTTCTTCCTCCCGAGACC		
SGO0176	*argH*	F	TCCATCATGCCACAAAAGAA	96	This study
		R	AACCGTCAGAAGGCTGAAGA		
SGO1103	*carA*	F	GACCTGGAAATCCGGAAGAT	114	This study
		R	CGTTAGCCATGGAGAAGAGC		
SGO1104	*carB*	F	CGCTAAGATTCCACGCTTTC	92	17
		R	TAGCCATGACTTCCCCTGTC		
SGO0611, SGO1706, SGO1956, SGO2129	16S rRNA gene	F	AGACACGGCCCAGACTCCTAC	138	17
		R	CTCACACCCGTTCTTCTCTTACAA		

*^a^* F is forward; R is reverse.

##### Determination of pH and Ammonia in Culture Supernatants

The concentration of ammonia in culture supernatants was measured with an ammonia assay kit (Sigma), as described previously (39, 40). *S. gordonii* DL1 and its derivative strains were grown at 37 °C for 24 h in CDM containing 0.1% galactose and 1 mm l-arginine, with growth monitored every 8 h by measurement of *A*_600_. pH values in the culture supernatants were determined with an electrode.

##### Biofilm Assay

*S. gordonii* cells (10^8^) were stained with hexidium iodide and aerobically cultured in CDM containing 0.025% glucose for 16 h using an 8-well saliva-coated LAB-TEK Chamber Slide System (Nalge Nunc International, Naperville, IL). For analysis of mixed biofilm formation, *S. gordonii*, an early colonizer of the tooth surface, was first cultured the same way as described above, washed gently twice by PBS, and then co-cultured with 1.5 × 10^7^ *F. nucleatum* cells labeled with 5-(and-6)-carboxyfluorescine succinimidyl ester, as described previously (5). The bacterial cells (1.5 × 10^8^) were then used in an arginine-supplemented mixed biofilm experiment. *S. gordonii-P. gingivalis*-mixed biofilms were also generated in the same manner. As for the *F. nucleatum* mono-species biofilm formation, FITC-labeled organisms (2.5 × 10^7^ cells) were cultured in PBS with specific metabolites for 24 h. Biofilms were observed with a Zeiss LSM 510 confocal laser scanning microscope (Carl Zeiss MicroImaging GmbH, Germany). Obtained images were analyzed using the Imaris 7.0.1 software package (BitplaneAG, Zurich, Switzerland), as described previously (38).

##### Statistical Analysis

All pairwise comparisons of groups were performed using a two-tailed *t* test. All comparisons of multiple groups except for growth curve analyses were performed using one-way analysis of variance with post hoc paired comparisons conducted with Dunnett's test. Comparative analyses of bacterial growth were performed using repeated-measure analysis of variance with post hoc paired comparisons with Dunnett's test. All statistical analyses were performed with SPSS version 22 (IBM Japan, Tokyo, Japan), and *p* values of 0.05 were considered to be statistically significant. Metabolite set enrichment analysis (MSEA) was performed as described previously (41).

## Results

### 

#### 

##### Functional Characteristics of ArcD in S. gordonii

**FIGURE 1.**
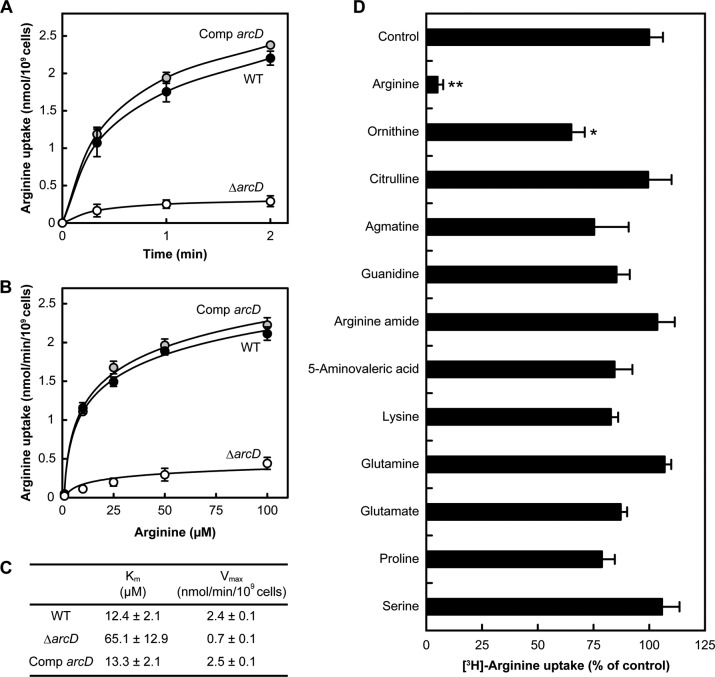
**Arginine uptake activity by ArcD of *S. gordonii*.** *A,* time course of radiolabeled arginine uptake by *S. gordonii* WT and its derivatives. The reaction was started by adding radiolabeled arginine to a final concentration of 50 μm. Data are shown as the mean ± S.E. of triplicate experiments. *B,* dose dependence of arginine uptake by *S. gordonii* WT and its derivatives after 1 min. Data are shown as the mean ± S.E. of triplicate experiments. *C,* kinetic parameters of arginine uptake by *S. gordonii* WT and its derivatives. *K_m_* and *V*_max_ values were calculated based on a Michaelis-Menten saturation curve with a nonlinear curve fit using ORIGIN 2015. *D,* effects of unlabeled arginine analogues and control amino acids on arginine uptake by *S. gordonii* WT after 20 s. The rate of arginine uptake was measured in the presence of candidate substrates added at 100-fold excess over radiolabeled arginine (50 μm). Data are shown as the mean ± S.E. of triplicate experiments. *, *p* < 0.05; **, *p* < 0.01 (*versus* control).

To investigate the function of ArcD in *S. gordonii*, we constructed an Δ*arcD* mutant and its complement strain (Comp *arcD*), and we assessed their uptake of exogenous ^3^H-labeled arginine. Consistent with a previous study of *Streptococcus pneumoniae* (37), arginine was rapidly incorporated by the *S. gordonii* wild-type strain (WT) within a few minutes (Fig. 1*A*). Although the Δ*arcD* mutant exhibited significant uptake reduction, complete inhibition was not observed. Kinetic analysis of arginine uptake revealed a *K_m_* value of 12.4 ± 2.1 μm and a *V*_max_ value of 2.4 ± 0.1 nmol/min/10^9^ cells for WT, although those were 65.1 ± 12.9 μm and 0.7 ± 0.1 nmol/min/10^9^ cells, respectively, for Δ*arcD*, and 13.3 ± 2.1 μm and 2.5 ± 0.1 nmol/min/10^9^ cells, respectively, for Comp *arcD* (Fig. 1, *B* and *C*). These results suggest that ArcD plays a major role in arginine uptake, although other minor arginine transport machineries exist in *S. gordonii*. To further examine the substrate specificity of ArcD, we measured the rate of arginine uptake by *S. gordonii* WT in the presence of unlabeled arginine analogues and control amino acids added at 100-fold excess over the radiolabeled arginine. The rate of arginine uptake was significantly inhibited by addition of unlabeled arginine (Fig. 1*D*). Ornithine also had a moderate effect on the inhibition of arginine uptake, although other arginine analogues (citrulline, agmatine, guanidine, arginine-amide, and 5-aminovaleric acid) and control amino acids (Lys, Glu, Gln, Pro, and Ser) had scant effects (Fig. 1*D*). These results suggested that ArcD-mediated arginine uptake has a high substrate specificity.

**FIGURE 2.**
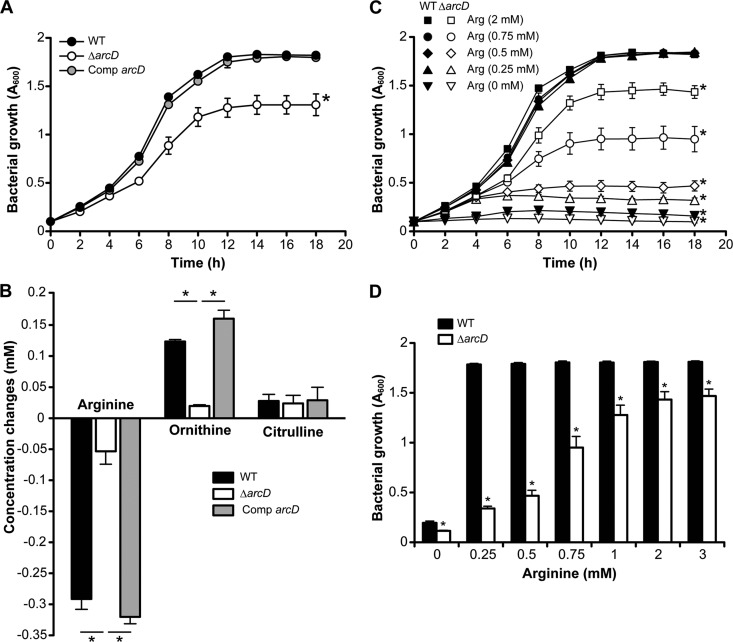
**Bacterial growth and shifts in extracellular concentrations of arginine, ornithine, and citrulline.** *A,* growth of *S. gordonii* WT and its derivatives in CDM containing 1 mm arginine and 1.2% glucose. Data are shown as the mean ± S.D. of triplicate experiments and are analyzed by repeated measures analysis of variance; *, *p* < 0.01 (*versus* WT or Comp *arcD*). *B,* arginine uptake and ornithine export by the tested strains. Shifts in the extracellular concentration of arginine and its related metabolites in culture supernatants were examined after 0 and 6 h of incubation. Data are shown as the mean ± S.E. of triplicate experiments. *, *p* < 0.01. *C,* growth of *S. gordonii* WT and Δ*arcD* in CDM containing various concentrations of arginine. Data are shown as the mean ± S.D. of triplicate experiments and analyzed by repeated measures analysis of variance. *, *p* < 0.01 (*versus* WT with 2 mm arginine). *D,* growth of *S. gordonii* WT and Δ*arcD* in CDM containing various concentrations of arginine after 6 h of incubation. Data are shown as the mean ± S.D. of triplicate experiments. *, *p* < 0.01 (*versus* WT at corresponding concentration).

To further characterize the function of *S. gordonii* ArcD, we inoculated the strains into CDM (pH 7.0) containing 1 mm arginine and 1.2% glucose, and we quantified intermediate metabolites in the ADS pathway in 6-h culture supernatants. The Δ*arcD* strain grew significantly slower than WT (Fig. 2*A*), and consumption of arginine and excretion of ornithine were also markedly decreased in Δ*arcD* (Fig. 2*B*). In contrast, no significant differences were observed in regard to extracellular citrulline concentrations. Bacterial growth in CDM with various concentrations of arginine was examined for 18 h, and the results showed that both WT and Δ*arcD* grew poorly in CDM without arginine (Fig. 2*C*). In the presence of 0.25 mm or more of arginine, WT showed fully recovered growth, whereas that of Δ*arcD* remained significantly reduced. Growth inhibition became diminished as the extracellular concentration of arginine increased (Fig. 2*D*), although no cultures of Δ*arcD* reached the final growth level of WT (Fig. 2, *C* and *D*). These findings suggest that ArcD is the most important protein for arginine uptake in *S. gordonii*, which may be crucial for growth of the organism, especially under a competitive environment with other arginine-utilizing microbes.

##### Contribution of ArcD to Global Arginine Metabolism in S. gordonii

**FIGURE 3.**
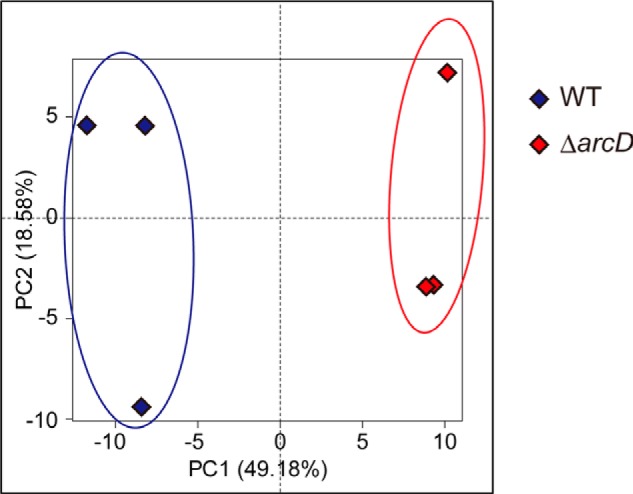
**Principal component analysis of intracellular metabolome of WT and Δ*arcD*.** Score plots for PC1 and PC2 are shown. *Blue* and *red circles* represent WT and Δ*arcD*, respectively.

The results presented above indicate that the activity of the arginine-ornithine antiporter ArcD is required for normal growth of *S. gordonii*, although another system likely compensates for arginine uptake, at least in part. To further evaluate the physiological consequences of the absence of ArcD protein, including attenuated ornithine efflux, we performed comprehensive profiling of the intracellular metabolites of both WT and Δ*arcD* using CE-TOF-MS. The ADS was previously reported to be induced by galactose (24); thus we cultured bacterial cells in CDM (pH 7.0) containing 0.1% galactose as the sole carbohydrate source. Our metabolomic analysis detected 233 metabolites, including 119 cationic and 114 anionic substances (supplemental Table S1). We then performed principal component analysis using the metabolomic data and found that the first principal component score (PC1) was related to *arcD* deletion (Fig. 3). Based on those principal component analysis findings, we conducted MSEA, which is able to identify which pathways are affected by lack of ArcD, as described previously (41). For this, factor loading in PC1 was used as an equivalent of the correlation coefficient between PC1 and each metabolite level. Statistical hypothesis testing for factor loading in PC1 was performed, and 86 metabolites were found to be significant at *p* < 0.05 (supplemental Table S2). Statistically significant pathways were selected by applying these metabolites to statistical hypothesis testing of cross-tabulation using Fisher's exact test in reference to a metabolite set list created based on the Kyoto Encyclopedia of Genes and Genomes. As shown in Table 3, ornithine and TCA cycles were identified as significantly enriched pathways in Δ*arcD* (*p* = 0.0110 and 0.0296, respectively). In contrast, glycolysis/glyconeogenesis and amino acid metabolism, such as Gly/Ser/Thr and Val/Leu/Ile, were significantly enriched in WT (*p* = 0.0211, 0.0004, and 0.0126, respectively), indicating that these pathways were significantly suppressed in Δ*arcD*.

**TABLE 3:** **Metabolite set enrichment analysis**

	Positive correlation with PC1		Negative correlation with PC1	
	*p* value*^a^*	*q* value	*p* value*^a^*	*q* value
Ornithine cycle	**0.0110**	0.2849	0.2958	0.5494
TCA cycle	**0.0296**	0.3850	1.0000	1.0000
Glutamate and glutamine metabolism	0.1253	1.0000	0.3466	0.5633
Histidine metabolism	0.1731	1.0000	0.8118	0.8442
Creatine metabolism	0.2414	1.0000	0.4237	0.6414
Lysine metabolism	0.2748	1.0000	0.6123	0.7580
Pyrimidine metabolism	0.2768	1.0000	0.6008	0.7580
Deoxyribonucleotide metabolism	0.3769	1.0000	0.1979	0.4890
Tryptophan metabolism	0.4260	1.0000	0.6697	0.7915
Polyamine metabolism	0.5012	1.0000	0.7505	0.8130
Tyrosine metabolism	0.5012	1.0000	0.7505	0.8130
Ribonucleotide metabolism	0.5611	1.0000	0.2251	0.4890
Shikimic acid metabolism	0.5669	1.0000	0.4441	0.6414
Purine metabolism	0.6835	1.0000	0.0610	0.3964
Alanine, aspartate, and asparagine metabolism	0.7175	1.0000	0.1440	0.4890
Glycolysis/glyconeogenesis	1.0000	1.0000	**0.0211**	0.1826
Pentose phosphate pathway	1.0000	1.0000	0.0913	0.4750
Valine, leucine, and isoleucine metabolism	1.0000	1.0000	**0.0126**	0.1633
Glycine, serine and threonine metabolism	1.0000	1.0000	**0.0004**	0.0092
Cysteine metabolism	1.0000	1.0000	0.1326	0.4890
Methionine metabolism	1.0000	1.0000	0.1518	0.4890
Proline metabolism	1.0000	1.0000	0.2445	0.4890
Beta-alanine metabolism	1.0000	1.0000	0.6123	0.7580
Taurine metabolism	1.0000	1.0000	0.3466	0.5633
Conjugated bile acid metabolism	1.0000	1.0000	0.2403	0.4890
Nicotinic acid metabolism	1.0000	1.0000	0.1979	0.4890

*^a^* Significant *p* values are in boldface type.

**FIGURE 4.**
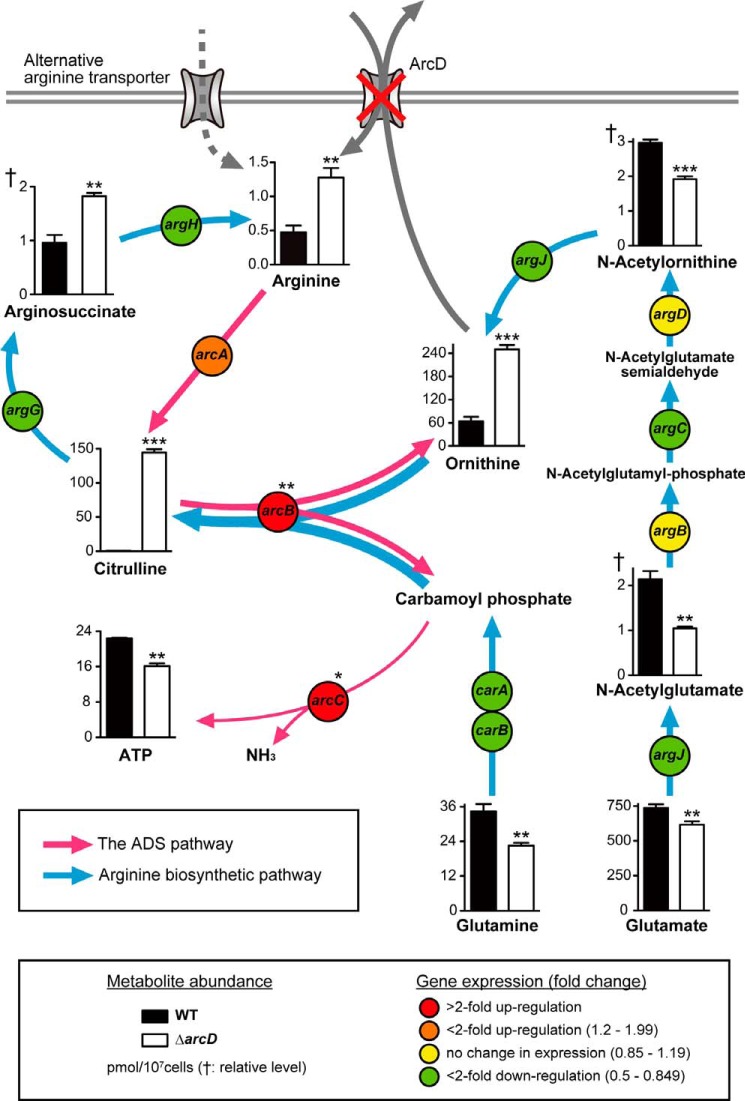
**Perturbed ornithine cycle in Δ*arcD*.** A schematic illustration of the metabolite abundance of *S. gordonii* involved in the ornithine cycle in combination with related transcriptional changes shown in the results of RT-PCR experiments is presented. Metabolites and transcripts were extracted following 8 h of incubation in CDM containing 1 mm arginine and 0.1 mm galactose at pH 7.0, followed by determination of those levels using CE-TOF-MS and real time RT-PCR, respectively. To minimize the influence of growth stage differences, cultures were started at an *A*_600_ value of 0.8. *Bars* represent the mean ± S.D. (*n* = 3). *, *p* < 0.05; **, *p* < 0.01; ***, *p* < 0.001; †, relative level of metabolite abundance.

Focusing on the amounts of intracellular metabolites involved in the ornithine cycle (Fig. 4), We observed that the amounts of the ADS intermediates arginine, ornithine, and citrulline were significantly increased in Δ*arcD* by 2.7-, 3.9-, and 310-fold, respectively (supplemental Table S1). In contrast, the amounts of *N*-acetylornithine and *N*-acetylglutamate, which are yielded during the early phase of the *de novo* arginine biosynthesis pathway passing from glutamate to ornithine, were significantly decreased by 1.7- and 2-fold, respectively (Fig. 4 and supplemental Table S1). This perturbation of the ornithine cycle in Δ*arcD* seemed to involve alternative arginine transporters and attenuated ornithine efflux, based on the arginine uptake and ultrapressure liquid chromatography assay results (Figs. 1 and 2*B*).

To gain further insight into the mechanisms leading to this perturbation, we assessed transcriptional changes in genes involved in arginine metabolism using real time RT-PCR. The relative transcriptional levels of *arcA*, *arcB*, and *arcC* in Δ*arcD* were enhanced by 1.6-, 2.7-, and 2.2-fold, respectively (Fig. 4 and Table 4). A previous study showed that the gene expression of *arcABC* is induced by a high arginine level (26), although we observed that the same gene regulation seemed to occur in Δ*arcD* because of increased internal arginine. Conversely, lack of ArcD suppressed the expression of genes encoding enzymes for *de novo* arginine biosynthesis, which catalyze the conversion of glutamate to ornithine (*argCJBD*), citrulline to arginine (*argGH*), and glutamine to carbamoyl phosphate (*carAB*) (Fig. 4). *S. gordonii* lacks anabolic ornithine carbamoyltransferase (ArgF; EC 2.1.3.3), which converts ornithine to citrulline, although previous reports have indicated that ArcB has potential for catalyzing this reaction (17, 42). Together, these findings suggest that the loss of ArcD causes accumulation of ornithine, leading to a halt of arginine catabolism as well as suppression of *de novo* arginine biosynthesis by end product inhibition or negative gene regulation. It is also likely that the ArcB-catalyzed conversion of ornithine to citrulline contributes to remarkable accumulation of citrulline. It should be also noted that Δ*arcD* showed a decrease in ATP levels despite up-regulation of the ADS genes (Fig. 4), indicating an ADS malfunction.

**TABLE 4:** **Relative changes in mRNA expression**

ORF	Gene	Relative fold expression in Δ*arcD vs.* WT*^a^*
SGO1593	*arcA*	1.650 ± 0.266
SGO1592	*arcB*	2.779 ± 0.266
SGO1591	*arcC*	2.299 ± 0.248
SGO1569	*argC*	0.775 ± 0.081
SGO1568	*argJ*	0.844 ± 0.106
SGO1567	*argB*	1.009 ± 0.124
SGO1566	*argD*	1.032 ± 0.094
SGO0175	*argG*	0.637 ± 0.069
SGO0176	*argH*	0.783 ± 0.075
SGO1103	*carA*	0.781 ± 0.088
SGO1104	*carB*	0.786 ± 0.071

*^a^* Values represent the mean ± S.E. (*n* = 3).

##### Growth and Ammonia Production of ΔarcD in Acidic Environment

**FIGURE 5.**
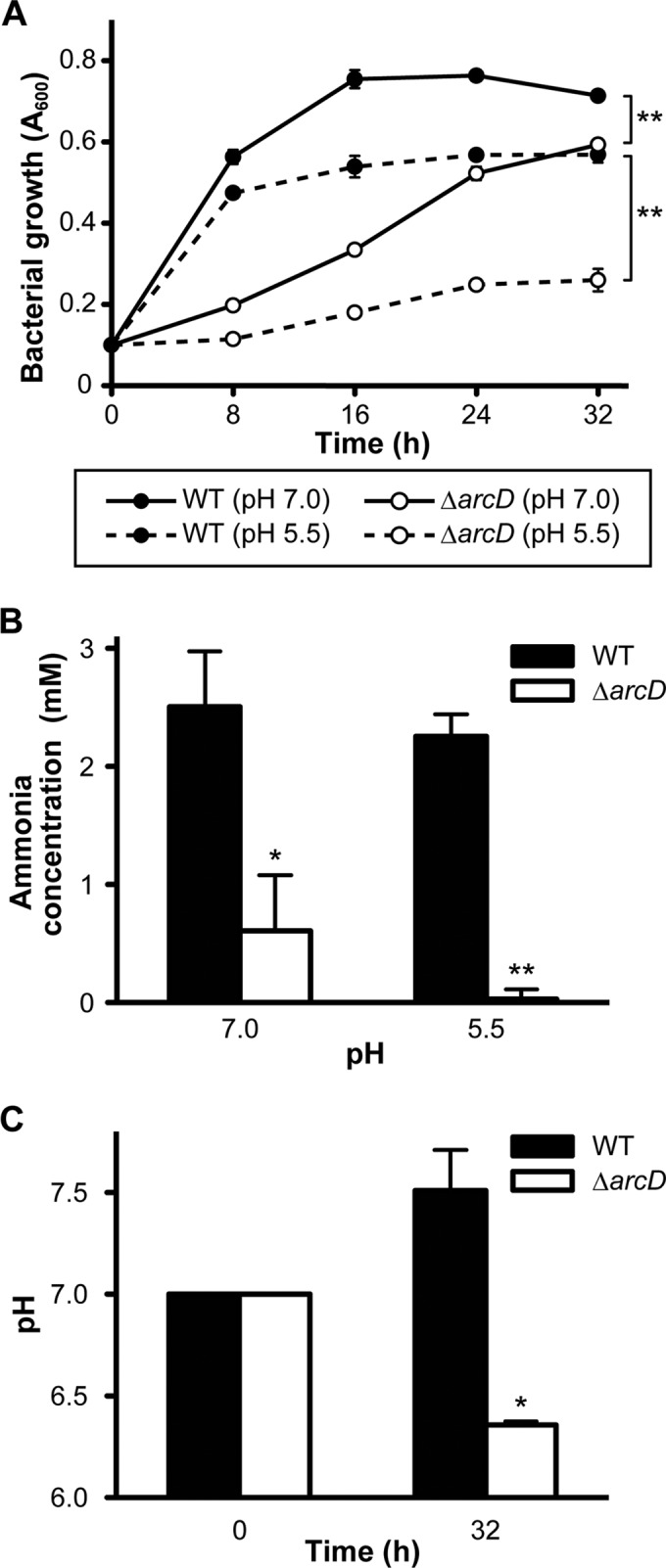
**Growth impairment of Δ*arcD* associated with reduced production of ammonia in both neutral and acidic environments.** *A,* growth of *S. gordonii* WT and *arcD* mutant in CDM containing 1 mm arginine and 0.1% galactose with pH values adjusted to 7.0 and 5.5. Data shown are representative of three individual experiments performed in triplicate and presented as the mean ± S.D. with analysis by repeated measures analysis of variance; **, *p* < 0.01. *B,* changes in amount of ammonia in medium following 32 h of incubation. Data are presented as the mean ± S.E. of a representative experiment performed in triplicate; *, *p* < 0.05; **, *p* < 0.01. *C,* medium pH following 32 h of incubation after initial adjustment to 7.0. Data are presented as the mean ± S.E. of a representative experiment performed in triplicate; *, *p* < 0.05.

Our metabolomics findings suggested that ammonia production is reduced in Δ*arcD* due to an ADS malfunction; thus, we assessed the contribution of ArcD to alkali production and acid tolerance in *S. gordonii*. First, we compared the growth kinetics of Δ*arcD* with those of WT in CDM containing 1 mm arginine and 0.1% galactose at pH 7.0 and 5.5. At pH 7.0, Δ*arcD* grew slower than WT and did not reach the same growth level even in the stationary phase. At pH 5.5, Δ*arcD* did not show any growth, although WT showed ∼70% growth as compared with that at pH 7.0 (Fig. 5*A*).

We also determined ammonia production and low pH tolerance in each strain. After 32 h of incubation, the amount of ammonia in the mutant culture supernatant was significantly reduced by 4.1- and 80-fold at pH 7.0 and 5.5, respectively, as compared with WT (Fig. 5*B*). The pH of the WT culture medium, initially adjusted to 7.0, increased to 7.5 ± 0.19, whereas that of the mutant culture decreased from 7.0 to 6.3 ± 0.017 (Fig. 5*C*). These results indicate that Δ*arcD* is more susceptible to pH stress than WT due to its reduced ability to antagonize milieu acidification. Thus, ArcD of *S. gordonii* is required for adequate function of the ADS to achieve optimal growth of the organism.

##### ArcD-mediated Ornithine Efflux Facilitates Community Development with F. nucleatum

**FIGURE 6.**
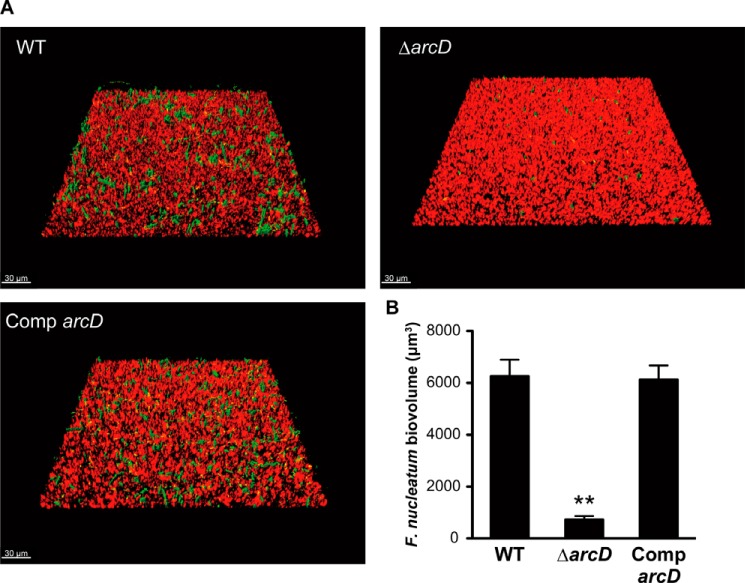
**Quantification of *F. nucleatum* accumulation in biofilms formed by various *S. gordonii* strains.** *A,* representative CLSM images showing typical architecture of biofilms following reconstruction by Imaris software. *S. gordonii* cells were stained with hexidium iodide (*red*), and biofilms were formed in the bottom chambers after 16 h of incubation, then gently washed with PBS, and co-cultured for 24 h with FITC-labeled *F. nucleatum* (*green*) suspended in PBS. *B,* biovolume of *F. nucleatum* after 24 h of co-incubation. Data shown are representative of three individual experiments and are presented as the mean ± S.E.; **, *p* < 0.01.

We next investigated whether *S. gordonii* ArcD is involved in bacterial metabolic interactions with other species. As a partner species, we focused on *F. nucleatum*, because interactions between it and *S. gordonii* have been shown to be affected by arginine and its derivatives (11, 33, 43). To this end, we assessed the ability of Δ*arcD* to form dual-species biofilms with *F. nucleatum* using confocal laser scanning microscopy (CLSM). *S. gordonii*, an early colonizer, was first cultured in CDM in saliva-coated glass bottom wells, followed by co-culturing with the succeeding colonizer *F. nucleatum* in PBS, as described previously (5). To quantify the biovolume of *F. nucleatum* in the resulting hetero-species biofilms, green fluorescent regions elicited by FITC were determined using Imaris software. Three-dimensional re-constructions of representative CLSM images are shown in Fig. 6*A. F. nucleatum* accumulation in Δ*arcD* biofilm was significantly decreased by 8.6-fold as compared with WT (Fig. 6*B*). In addition, complementation of ArcD completely restored the fusobacterial biovolume. As a control experiment, *S. gordonii* mono-species biofilms were also analyzed, which showed that the biovolume of biofilm formed by Δ*arcD* was nearly equivalent to that of the WT biofilm (data not shown). These results suggest that ArcD is involved with dual-species biofilm formation with *F. nucleatum*, although it has negligible influence on *S. gordonii* sole-species biofilm.

**FIGURE 7.**
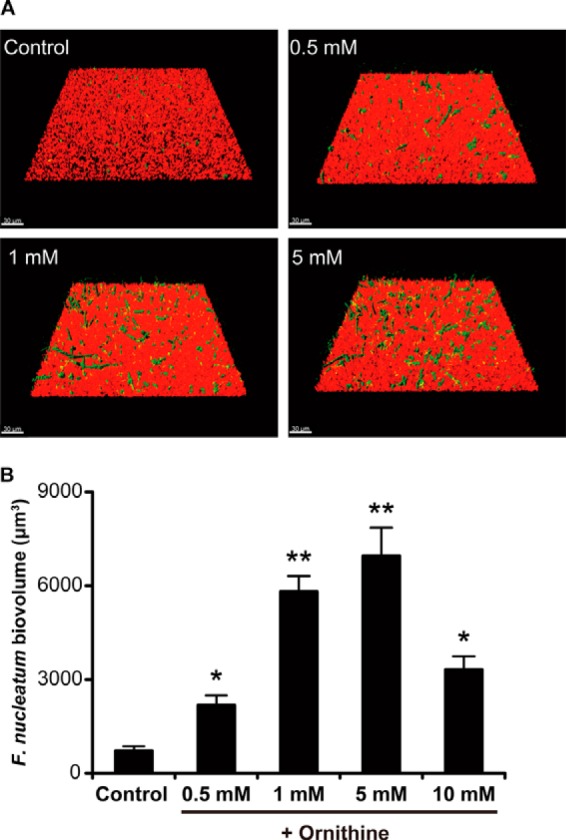
**Effect of ornithine supplementation on *F. nucleatum* accumulation in biofilms formed in co-cultures with *S. gordonii* Δ*arcD*.** *A,* representative images of typical biofilm architecture. *S. gordonii* (*red*), *F. nucleatum* (*green*) dual-species biofilms were formed in the presence of various concentrations of ornithine after 24 h of co-incubation. *B,* biovolume of *F. nucleatum* after 24 h of co-incubation. Data shown are representative of three individual experiments and presented as the mean ± S.E.; *, *p* < 0.05; **, *p* < 0.01.

Arginine-inhibited co-aggregation of *F. nucleatum* with streptococcal species is mediated by RadD (11). To evaluate the effect of arginine on *S. gordonii-F. nucleatum* biofilm formation, we performed cultures in the presence of 1 mm arginine. However, no significant difference in regard to biovolume was observed (data not shown). Based on our speculation that ornithine exported by ArcD would facilitate the development of *S. gordonii-F. nucleatum* biofilm, we analyzed the biovolume of biofilms formed by *F. nucleatum* co-cultured with Δ*arcD* in the presence of various concentrations of ornithine. At low concentrations, ornithine restored fusobacterial accumulation in the biofilm in a dose-dependent manner, which reached a peak with 5 mm ornithine (Fig. 7).

**FIGURE 8.**
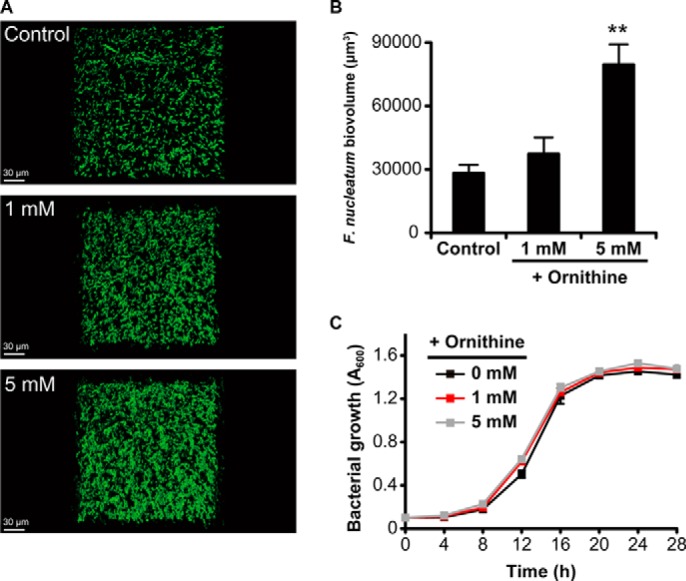
**Effect of ornithine supplementation on mono-species biofilm formation by *F. nucleatum*.** *A,* representative images of typical biofilm architecture. Cells were stained with FITC (*green*), and mono-species biofilms were formed in the presence of various concentrations of ornithine after 24 h of incubation. *B,* biovolume of *F. nucleatum* after 24 h of incubation. Data shown are representative of three individual experiments and presented as the mean ± S.E.; **, *p* < 0.01. Images were acquired by CLSM and reconstructed by Imaris software. *C,* planktonic growth of *F. nucleatum* in the presence of various concentrations of ornithine. Data are shown as the mean ± S.D. of triplicate experiments.

We also investigated the direct effect of ornithine on *F. nucleatum* mono-species biofilm, and found that additional ornithine (1 and 5 mm) increased the biovolume in a dose-dependent manner, with significant increases observed at 5 mm (Fig. 8, *A* and *B*). Meanwhile, ornithine supplementation in growth medium only modestly enhanced the planktonic growth of *F. nucleatum* (Fig. 8*C*). These results indicate that ArcD-mediated ornithine export facilitates *S. gordonii-F. nucleatum* heterotypic biofilm formation.

**FIGURE 9.**
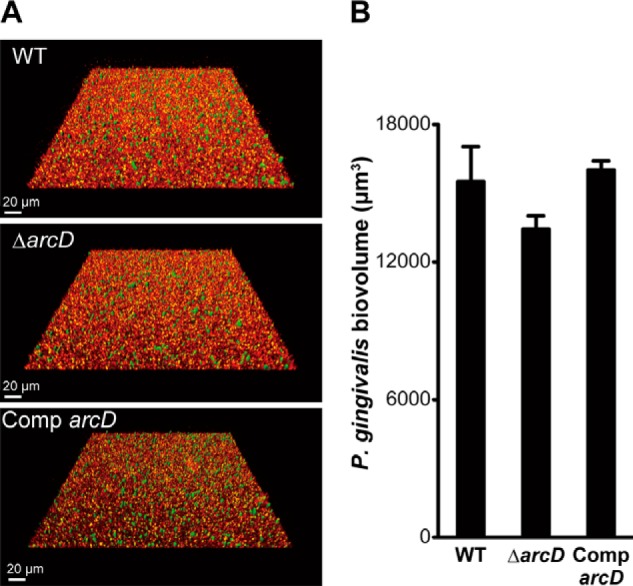
**Quantification of *P. gingivalis* accumulation in biofilms formed by *S. gordonii* strains.** *A,* representative images of typical biofilm architecture. *S. gordonii* biofilms (*red*) were formed after 16 h of incubation, then gently washed with PBS, and co-cultured for 24 h with FITC-labeled *P. gingivalis* (*green*) suspended in PBS. *B,* biovolume of *P. gingivalis* after 24 h of co-incubation. Data shown are representative of three individual experiments and presented as the mean ± S.E.

Finally, we examined whether the loss of ArcD affects dual-species biofilm formation with *P. gingivalis* (Fig. 9). In contrast to *F. nucleatum*, no significant difference in *P. gingivalis* accumulation (*p* > 0.05) could be discerned whether it is grown in biofilms of *S. gordonii* of the WT, Δ*arcD*, or complemented *arcD* strains. These results indicate that ArcD-mediated bacterial interaction has species selectivity.

## Discussion

In this study, we found that *S. gordonii* ArcD is a key tool not only for arginine uptake and ornithine excretion to maintain the homeostasis of arginine metabolism but also for metabolic interaction with *F. nucleatum* in biofilm community development. *S. gordonii* has been recognized as an accessory pathogen due to its ability to support periodontitis-related species, including metabolic cooperation, in the context of heterotypic communities (15). The present findings provide further evidence in that regard, as cross-feeding between oral streptococci and *F. nucleatum* has not been reported previously. Moreover, we demonstrated that ArcD modulates alkali and energy production in conjunction with the ADS in *S. gordonii*, and it maintains the homeostasis of arginine metabolism through ornithine efflux.

Although we did not find evidence of a direct exchange activity of arginine and ornithine, we speculated that ArcD of *S. gordonii* serves as an arginine-ornithine antiporter (Figs. 1 and 2*B*), similar to other microbes (29, 39, 44), and that its activity is finely regulated, because excessive extracellular ornithine inhibited the arginine uptake (Fig. 1*D*). However, another relatively inefficient arginine uptake system likely exists in *S. gordonii*, because arginine supplementation only partially restored the growth of Δ*arcD* in a dose-dependent manner (Fig. 2, *C* and *D*). Notably, growth impairment of Δ*arcD* was most pronounced at low concentrations of arginine (Fig. 2, *C* and *D*). Mature dental biofilm reportedly contains ∼0.2 mm arginine (45), thus the potent arginine uptake activity mediated by ArcD may play a critical role for survival of *S. gordonii* when in a biofilm-forming state.

*S. gordonii*, abundant in healthy supra- and subgingival biofilms, has long been considered beneficial to the host largely because of its ability to moderate biofilm acidification through the ADS (46). The present results suggest that ArcD is required for optimal functioning of the ADS by providing arginine, which prompts not only the production of ammonia that neutralizes acidification of the surroundings (Fig. 5), but also the production of ATP necessary for cell division in the exponential phase (Fig. 4). A previous study noted that the ADS in *S. gordonii* had higher susceptibility to carbon catabolite repression than that in other closely related oral streptococci, indicating its primary involvement in energy generation under a glucose-depleted condition, such as seen in the subgingival region (47). Thus, ArcD and ADS functions are likely more crucial for *S. gordonii* organisms localized in subgingival areas of the oral cavity as compared with those in supragingival areas.

Results from MSEA showed that TCA and ornithine cycles were significantly enriched in Δ*arcD* (*p* < 0.05), whereas glycolysis and amino acid metabolism in Δ*arcD* were significantly suppressed (*p* < 0.05) (Table 3). Nitrogen starvation of *E. coli* has been shown to cause a similar metabolic shift in the TCA cycle, glycolysis, and amino acid biosynthesis (48, 49), suggesting a nitrogen deficiency in the present Δ*arcD* and that *S. gordonii* depends on arginine supplied by ArcD as a main nitrogen source. The most impacted metabolic pathway by *arcD* deletion was shown to be the ornithine cycle, which includes the ADS and arginine *de novo* biosynthesis pathway. The former has been extensively studied in oral streptococci (46), although little is known about the arginine biosynthesis pathway in those species.

A variety of streptococci must acquire arginine from an extracellular source, because the *de novo* arginine biosynthetic pathway is incomplete (50). Nevertheless, a few oral streptococci, including *S. gordonii,* harbor a complete set of genes related to that pathway, which are annotated in Kyoto Encyclopedia of Genes and Genomes. Despite this, activation of arginine biosynthesis is not efficient in *S. gordonii*, possibly due to the absence of the annotated gene encoding an anabolic ornithine carbamoyltransferase (ArgF; EC 2.1.3.3), which is responsible for one-way conversion of ornithine to citrulline (17). Previous reports have indicated that the reaction of ArcB in *Pseudomonas aeruginosa* is bilateral and favors citrulline synthesis from a thermodynamic perspective (42). Moreover, the amino acid sequence of *S. gordonii* ArcB shows high similarity to that of *Lactococcus lactis* ArgF (17). More recently, ArcB was shown to be essential for arginine biosynthesis in *S. gordonii* (51). Therefore, ArcB in *S. gordonii* is likely able to catalyze the conversion of ornithine into citrulline when the *de novo* arginine biosynthesis pathway is active and vice versa when an ADS-mediated arginine catabolic reaction is activated.

Data from metabolomics analysis revealed higher levels of intermediates in the ADS pathway, although those were lower in the arginine biosynthetic pathway in the mutant strain (Fig. 4). These results suggest that loss of ArcD causes failure of ornithine efflux and intracellular ornithine accumulation, leading to a halt of arginine catabolism and suppression of the early phase of *de novo* arginine biosynthesis. This notion appears to be well supported by previous findings showing that loss of the succinate exporter in *Corynebacterium glutamicum* led to intracellular accumulation of succinate, resulting in inhibition of the upstream pathway of sugar metabolism (52). Specifically, extreme citrulline accumulation in Δ*arcD* may be explained by ornithine accumulation, thus allowing ArcB to catalyze the conversion of ornithine to citrulline. The unexpectedly high level of arginine shown in the mutant strain was probably due to a constant, albeit relatively slow, uptake of arginine by an alternative arginine transporter as well as the halt of arginine metabolic processes caused by ornithine accumulation. Results from real time RT-PCR experiments largely correspond to metabolic shifts observed in Δ*arcD*, although they seem contradictory to the finding of reduction of ATP and NH_3_, end products of the ADS, because expression levels of the *arcABC* genes were increased. One interpretation is that higher levels of intracellular arginine promote the expression of *arcABC* and activate ArcB-catalyzed conversion of ornithine to citrulline, resulting in depletion of carbamoyl phosphate, a substrate of ArcC. This deprivation of carbamoyl phosphate is likely to suppress production of the ADS end products ATP and NH_3_. Taken together, these findings suggest that ArcD-mediated ornithine export contributes to not only initiation of *de novo* arginine biosynthesis, but also optimization of the ADS by ensuring that ArcC catalyzes the conversion of carbamoyl phosphate to ATP and NH_3_. Ornithine may be a key regulator for balancing the ADS with *de novo* arginine biosynthesis in *S. gordonii*, although further studies are needed to elucidate how the global arginine metabolism is regulated in this organism.

The most striking finding in this study was a significant reduction of *F. nucleatum* accumulation in biofilm formed by the *S. gordonii* Δ*arcD* mutant (Fig. 6). Ornithine supplementation restored the biovolume of *F. nucleatum* in mono-species biofilms as well as dual-species biofilms with the *S. gordonii* Δ*arcD* mutant (Figs. 7 and 8), suggesting that ArcD-mediated ornithine efflux is indispensable for successful colonization by *F. nucleatum*, leading to *S. gordonii-F. nucleatum* community development. However, provision of ornithine only modestly enhanced the planktonic growth of *F. nucleatum*, suggesting that ornithine cross-feeding is important for the biofilm-forming state of this organism. It should be noted that the loss of ArcD did not affect the interaction between *S. gordonii* and *P. gingivalis* (Fig. 9), which may be indicative of metabolic selectivity in the oral microbial community.

*F. nucleatum* has been referred to as a “bridging organism” that links early and late colonizers of oral microbial communities, and it contributes to the pathogenicity of periodontitis (9, 12, 14). This organism also has a preference for amino acids, although glutamate in particular has been speculated to be the major energy source for this species (53, 54). Notably, the *F. nucleatum* subspecies *nucleatum* ATCC 25586 showed significant uptake efficiency of ornithine in an examination of related fusobacterial species (55). In addition, a previous study that used a multispecies proteomics approach found that *F. nucleatum* exhibited a significant increase in protein expression level of ornithine decarboxylase, an enzyme responsible for conversion of ornithine/arginine to putrescine, in community biofilm formed with *S. gordonii* (43). Therefore, this pathogen seems to utilize ornithine released by *S. gordonii* ArcD as a substrate of ornithine decarboxylase. We also confirmed that supplementation with 5 mm putrescine enhanced monospecies biofilm formation by *F. nucleatum* (data not shown), and we are currently investigating the effects of putrescine and other polyamines on the physiology of *F. nucleatum*.

*F. nucleatum* also possesses RadD, which is responsible for adherence to oral streptococci, and RadD-mediated adhesion was shown to be inhibited by arginine in co-aggregation assay results (11). Here, we examined the effect of extracellular arginine on dual-species biofilm formation to exclude the possibility that reduced accumulation of *F. nucleatum* in Δ*arcD* biofilm is due to an elevated concentration of arginine sufficient to inhibit the adhesion activity of RadD, based on the assumption that *F. nucleatum* releases arginine. We found that extracellular arginine did not have an influence on dual-species biofilm formation. Therefore, a reduction of *F. nucleatum* in Δ*arcD* biofilm is unlikely to involve RadD inhibition. In contrast, it seems that ArcD elevates *S. gordonii-F. nucleatum* symbiosis by lowering the level of extracellular arginine to enhance the adhesive properties of RadD as well as by excreting ornithine to attract *F. nucleatum*.

To the best of our knowledge, there are few reports of microbial metabolic interactions with *F. nucleatum*. Short-chain fatty acids produced by *F. nucleatum* as a metabolic by-product were shown to promote *A. naeslundii* biofilm formation (56), although a more recent study suggested that propionic acid produced by *F. nucleatum* underpins the colonization of *Neisseria meningitidis* in the adult oral cavity (57). However, studies to date on fusobacterial metabolic interactions were largely centered on short-chain fatty acids, and our findings are the first reported evidence that *F. nucleatum* utilizes amino acids produced by other bacteria. Because *F. nucleatum* has also gained attention as an important pathogen because of its possible involvement in a diverse range of human pathologies, including colorectal cancer and preterm birth (58–61), our results provide insight into the physiology of this multifaceted species.

In addition, these findings provide additional information regarding the role of the ADS of oral streptococci in periodontitis. A recent proteomic study found that levels of ArcA, ArcB, and ArcC of *S. gordonii* were reduced in community biofilms formed with *P. gingivalis* as compared with mono-species biofilms, although the level of ArcD protein was below the limit of detection (33). However, that study also showed that community biofilms formed with *F. nucleatum* had markedly increased levels of ArcA, ArcB, and ArcC despite a significantly reduced level of ArcD in *S. gordonii*. Co-aggregation with *A. oris* was also reported to repress *arcD* gene expression in *S. gordonii* as compared with a mono-species state, suggesting that *S. gordonii* regulates arginine uptake by altering the ArcD expression level in response to competitive or compatible partner species in multispecies communities (17). Nevertheless, additional studies are needed to reveal the mechanisms underlying the regulation of this system in response to interactions with periodontitis-associated species.

In summary, we demonstrated that ArcD is required for maintaining homeostasis of the global arginine metabolism in *S. gordonii*. This study also identified a novel metabolic interaction that occurs between early and intermediate colonizers in community development, which may be the first step of a dysbiotic shift to periodontitis.

## Author Contributions

A. S., M. K., and E. H. carried out the metabolome analysis. A. S. did the MSEA. H. T. performed the transcriptional analysis. A. S. and M. K. carried out the community construction and analysis by confocal microscopy. A. S., H. T., and M. K. constructed the *arcD* mutant and the complement strains. M. K. and A. S. conceived the experiments. A. S., M. K., and A. A. wrote the manuscript.

## Supplementary Material

Supplemental Data

## Footnotes

3 The abbreviations used are:

ADS: arginine deiminase system
THB: Todd-Hewitt broth
CDM: chemically defined medium
CE-TOF-MS: capillary electrophoresis time-of-flight mass spectroscopy
MSEA: metabolite set enrichment analysis
CLSM: confocal laser scanning microscopy
PC1: first principal component score.
