# Supplementary material for: Arginine-Ornithine Antiporter ArcD Controls Arginine Metabolism and Interspecies Biofilm Development of Streptococcus gordonii
Source: J Biol Chem. 2015 Jun 17;290(35):21185–98. doi: 10.1074/jbc.M115.644401 (PMC4571851; doi:10.1074/jbc.M115.644401)
Supplement: Supplemental Data [file supp_290_35_21185__index.html]

Arginine-ornithine Antiporter ArcD Controls Arginine Metabolism and Interspecies Biofilm Development of Streptococcus gordonii — Arginine-Ornithine Antiporter ArcD Controls Arginine Metabolism and Interspecies Biofilm Development of Streptococcus gordonii — Ornithine Cross-feeding by S. gordonii ArcD to F. nucleatum — Supplemental Data 

# Arginine-Ornithine Antiporter ArcD Controls Arginine Metabolism and Interspecies Biofilm Development of *Streptococcus gordonii*

## Supplemental Data

- Supplemental Table S1 (.xlsx, 112 KB) - Metabolomic profile of detected 233 major metabolites
- Supplemental Table S2 (.xlsx, 25 KB) - Statistical hypothesis test of factor loading in PC1
